# PGRMC Proteins Are Coming of Age: A Special Issue on the Role of PGRMC1 and PGRMC2 in Metabolism and Cancer Biology

**DOI:** 10.3390/cancers13030512

**Published:** 2021-01-29

**Authors:** Michael A. Cahill, Hans Neubauer

**Affiliations:** 1 School of Biomedical Sciences, Charles Sturt University, WaggaWagga, NSW 2678, Australia; 2 ACRF Department of Cancer Biology and Therapeutics, The John Curtin School of Medical Research, Canberra, ACT 2601, Australia; 3 Department of Gynecology and Obstetrics, University Women’s Hospital of Dusseldorf, 40225 Duesseldorf, Germany

## 1. PGRMC Research History

This is a preface by the guest editors of the special issue of *Cancers* featuring the biology of progesterone (P4) receptor membrane component (PGRMC) proteins as it relates to metabolism and cancer. PGRMC proteins are members of the cytochrome b5-related membrane-associated progesterone receptor (MAPR) family, of which humans have four members: PGRMC1, PGRMC2, Neudesin and Neuferricin [1,2].

In the early 2000s, we were both part of a German Human Genome Project (Proteomanalyse von Brustkrebsgenen, 01 KW0102, 2001–2004) that focused on the development of highly sensitivity proteomics methods for the identification of proteins whose properties were altered between clinical protein samples. Before the rise and supremacy of mass spectrometric proteomics methods, we developed an advanced gel electrophoresis and highly sensitive/accurate differential detection method to examine cryoconserved breast cancer tissues, comparing estrogen receptor positive versus negative patient cohorts [3,4]. Of the many hundreds of proteins identified (and manually biologically assessed in those days), we detected three differently charged isoforms of PGRMC1 [4].

The background search for a patent application [5] revealed that the protein had been identified from numerous different organisms in perplexingly different biological contexts. These included roles inducing non-genomic P4-responses in tissues of the female reproductive tract, in axon guidance during the embryological formation of the central nerve cord, in cytochrome P450 (CYP450)-mediated steroidogenesis in the adrenal cortex, as a component of the SREBP/Insig/SCAP complex that regulates the mevalonate pathway and lipid synthesis, and as a stress response protein which affects cellular life/death probabilities [6,7,8]. In 2011, the question was raised if the increase in breast cancer observed in the estrogen plus progestin arm of the Women’s Health Initiative trial can be explained by PGRMC1 [9,10]. Intriguingly, there were no obvious connections between many of these functions for this small cytochrome b5-domain heme-binding protein. It seemed to potentially occupy a regulatory nexus position in cell biology [4,11].

We had identified several phosphorylated residues from data base analyses. By phosphatase analysis, we demonstrated that the three identified PGRMC1 isoforms from human breast cancer patients differed in phosphorylation status (to our knowledge the first identification of cancer relevant phosphorylations from clinical samples). When we mutated the phosphorylation sites, we changed the ability of cancer cells to survive toxic treatments [4]. We also noted that PGRMC1 induction preceded that of the Hif1-induced GLUT1 glucose transporter in the hypoxic zone surrounding the necrotic core of ductal carcinoma in situ lesions, leading us to hypothesize that PGRMC1 could be involved in the cancer-specific switch to glycolytic biology even in the presence of oxygen (aerobic glycolysis), known as the Warburg effect [4]. This was the most interesting protein that we had encountered, and its mysterious allure has occupied both of our subsequent research careers.

## 2. Recent Advances and Current Status

In the more than 15 years that have elapsed since our PGRMC1 journey began, of course we were not the first nor have we been alone. The increasing interest in the PGRMCs led to a constant increase of publications in ‘PubMed’ to currently around 356 reports [‘PGRMC1 OR PGRMC2’] (December 2020). The majority of these are associated with ‘metabolism’ (283), ‘cancer’ (138), and ‘progestin’ (120). These numbers not only indicate the current focus of the ‘PGRMC-research’, but also reflect a substantive connection between these fields. Metabolic changes such as alterations in lipid- and cholesterol-associated pathways mediated by PGRMCs not only enhance obesity [12], but are also a recognized hallmark of cancer (cancer-associated metabolic programming) [13] associated with potential therapy resistance mechanisms [14].

The field has been substantially advanced by the development of a cre/lox tissue-targetable mouse knockout system for PGRMC1 and PGRMC2, which have demonstrated roles in reproductive tissues [15,16,17,18], and in heme trafficking by PGRMC2 from mitochondria to nucleus [19]. Zebrafish knockouts produce analogous results [20]. A separate N-terminal frameshift mutation knockout of mouse PGRMC1 has revealed roles in modulation of hepatic fatty acid synthesis [21], mammary gland development [22], and regulation of hepatic glycolysis/gluconeogenesis [23]. The PGRMC1 crystal structure has provided substantial insight into PGRMC1 biology, its probable role as a sensor of gases, such as CO and NO, and the relationship of these to the redox state of heme iron [24,25].

The small molecule AG-205 inhibitor was designed against a plant MAPR protein [26], and was shown to modulate PGRMC1-related tumor growth [27], tumor cell survival [28], and EGFR levels [29]. Many subsequent studies have gone on to use AG-205 as a ‘PGRMC1-specific’ inhibitor (it is marketed as such, but let the buyer beware), although this was never demonstrated or claimed. Having been generated against a plant MAPR protein, very distantly related to mammals, AG-205 could conceivably bind to all of the human MAPR proteins, or perhaps to other targets. While identifying proteins whose presence in PGRMC1 protein complexes was affected by AG-205 (many of which were related to actin biology), we observed marked PGRMC1-independent effects of AG-205 [30]. Perhaps unsurprisingly, Eckhardt’s group showed conclusively that AG-205 induced the formation of large cytoplasmic membranous structures in cells devoid of PGRMC1 and PGRMC2 [31]. Clearly, while AG-205 does affect PGRMC1 biology, it is not ‘PGRMC1-specific’ and many published conclusions should be revisited.

The association of PGRMC1-like proteins with steroidogenic lanosterol-14-demethylase CYP450 enzymes from yeasts to mammals was recognized early [32,33], and is extended by PGRMC interaction with multiple CYP450s in humans that catalyze a diverse range of reactions, including the inactivation of chemotherapeutics [7,24,34,35,36,37,38]. Steroid responses are central to reproductive tissues, and PGRMCs are associated with both healthy and cancerous vertebrate reproductive biology. Interestingly, the effects manifest as diversely as the regulation of the spindle-mediated meiotic/mitotic separation of chromosomes, induction of cell and tissue maturation processes, and cancer survival [17,20,22,39,40,41,42,43,44,45,46,47,48,49,50,51,52].

Much of the pregnancy-related P4 response is mediated by the classical (nuclear transcription factor) P4 receptor (PGR) [44]. However, in some cells, including the granulosa feeder cells of the oocyte, the P4 response is mediated solely by PGRMC proteins [17]. Whereas the nuclear transcription factor steroid receptors arose in eumetazoans, and became diversified in chordates, the PGRMC/MAPR steroid response is probably far older [53].

Analogously to the situation with a lack of ‘PGRMC1-specific’ inhibition by AG-205, the compound mifepristone (RU486, most famous as an abortion drug) was until recently thought to be a specific antagonist of the PGR. It has now been shown to also act through PGRMC1 and non-canonical TGFβ1 signaling [54,55], reinforcing the proposed centrality of steroid biology to PGRMC1 function [6,56,57].

PGRMC1 is present in the complex with SREBP/Insig/SCAP that regulates mevalonate pathway leading to sterol synthesis (SREBP1 and SREBP2), or modulates fatty acid synthesis (SREBP1) [21,58,59,60,61,62]. Together with the regulation of lanosterol-14-demethylase by PGRMC like proteins from yeast to mammals [32,33] (which is the modification of the first sterol produced in each of these groups), and the involvement of PGRMC1 in mediating steroid responses, we proposed that steroid biology may be central to PGRMC1 function, where it could act as a feedback regulatory nexus central integrator [4,6].

Somehow, this seemed to be related to mitochondrial function [56], and we showed that PGRMC1 phosphorylation status can exert the most dramatic effects on mitochondrial form (fission versus fusion) and cell metabolism. This involved not just mitochondria, but also the PI3K/Akt pathway via tyrosine 180 [63]. PGRMC1 modulation of the Akt pathway has been reported multiple times [4,64,65]. Mitochondrial PGRMC1 associates with and regulates ferrochelatase, the last and rate-limiting reaction in heme synthesis [66], and as mentioned above, PGRMC2 acts as a chaperone in the transport of heme from mitochondria to the nucleus, where the heme results in the up-regulation of genes whose protein products are mitochondrial [19].

Altered mitochondrial biology was also associated with PGRMC1-driven changes in glycolysis [63], concordant with our earlier hypothesis that PGRMC1 could be associated with the Warburg effect. In 2019, Sabbir independently demonstrated that HEK293 cells induced aerobic glycolysis upon P4 treatment, which involved altered abundance of post-translationally modified PGRMC1 isoforms. A 70 kDa endoplasmic reticulum/mitochondrial species was proteasomally degraded, and the abundance of a 100 kDa nuclear species was greatly reduced upon P4 treatment [67].

PGRMC1 has long been known to occupy multiple subcellular locations, from endoplasmic reticulum, cytoplasmic soluble, plasma membrane, nucleus/nucleolar, and mitochondrial [6,57]. It has been proposed that post translational modifications would regulate localization, including phosphorylation, ubiquitination, and Sumoylation. In particular, Sumoylation was thought to be associated with PGRMC1 translocation to the nucleus [46], which was confirmed by Sabbir’s documentation of P4-induced changes in PGRMC1 phosphorylation, ubiquitination, and Sumoylation, with Sumoylated isoforms associated with nuclear fractions [67]. It will be interesting to see whether Sumoylation of heme-chaperoning PGRMC2 is associated with its nuclear localization, and to what extent PGRMC1 and PGRMC2 functions overlap or differ.

The dramatic mitochondrial and metabolic changes induced by PGRMC1 phosphorylation mutants were accompanied by changes in genomic CpG epigenetic methylation status, which associated PGRMC1 activity with stem cell biology and control of differentiation status [68]. That suggests that PGRMC1 is involved in an ancient regulatory mechanism which regulates epigenetic genomic packaging and basic cellular metabolic strategies. This may mechanistically involve steroidogenesis, which can alter membrane properties or activate specific receptors to induce cytoplasmic or nuclear responses. Alternatively, steroidogenesis could modulate other cell biological functions.

Riad et al. strengthened the association between PGRMC1 and steroid biology by demonstrating that a complex containing PGRMC1/TMEM97 and the LDL receptor is responsible for a mechanism of accelerated LDL import [69]. The mechanism has been confirmed in embryonic fibroblast 3T3L1 cells during differentiation to adipocytes [12]. Because cancer cells often require elevated cholesterol levels, and hepatically-synthesized cholesterol is transported via the blood using the LDL system, this pathway is thought to be cancer-relevant [69].

In a subsequent publication Riad et al. have shown that the same PGRMC1/TMEM97/LDLR complex mediated the endocytic uptake of amyloid beta (Aβ) 42 [70]. Oligomers of Aβ42 binding to synapses are involved in synaptic dysgenesis and cognitive decline in Alzheimer’s disease [71,72,73,74] and this process involves PGRMC1-dependent membrane trafficking [75,76]. MAPR proteins including PGRMCs play many roles in the central nervous system, involving steroid responses, cytoskeletal regulation, metabolic control, survival and stress recovery, and of course cancer [2,7,25,35].

## 3. Why a Special Issue Now?

There are already a plethora of data and researchers working on the PGRMCs, with ever more details of their functions being revealed. Yet, PGRMCs’ clinical utility appears to be under-appreciated (PGRMC researchers are constantly underfunded). Therefore, by highlighting different aspects of PGRMCs in this special issue we hope to sow the seeds towards revealing pathways that will lead to better characterization and exploitation of the clinical utility of PGRMCs.

This intention is also mirrored in the selection of the two editors. Both believe that this endeavor may be accomplished by highlighting recent findings on PGRMC functions in the context that these proteins were quintessentially enabling for the evolutionary development of the animal lineage leading to our species. This offers a hitherto unparalleled opportunity to collate, express, and consider the implications of recent findings for the field. The pieces of the PGRMC jigsaw puzzle need to be crafted, ordered, and placed into the context of the emergent greater picture. This stratospheric scale of ambition sets the stage for us to welcome contributions of the highest standard.

## 4. Fields of Research Interest

The fundamental understanding that PGRMC proteins exhibit so many different functions in so many functional contexts because of their foundational role in the evolutionary history of vertebrates provides a contextual perspective from which future advances can be conceptualized and brought to fruition. While this is a cancer-specific journal, we are not interested in only cancer manuscripts. The mechanisms that alter cell metabolism of any cell type could be relevant to cancer, just as understanding the origins of those mechanisms can illuminate and embellish our humanized rational understanding of them (involving cognitive processes that doubtlessly involve PGRMCs). Therefore, we will even consider manuscripts that are only tangentially related to cancer biology, if they could be relevant to tumor biology.

## 5. What We Hope to Achieve

In bringing together the most eminent international researchers in this important field in one timely special issue, we are hoping to coalesce concepts and accelerate the pace of progress in what we believe to be this centrally important field of research. We hope that this special issue can serve as a crucible for the coalescence of new discoveries that serves a catalytic function in generating new and exciting research directions. The meeting of leading minds so enabled should provide an aspirational forum for ongoing advances.

## 6. Manuscripts Invited

Therefore, we formally invite the world’s most talented researchers in the field of PGRMC biology to participate in this exciting and important endeavor. Contributions are eagerly anticipated.
